# USING INTERVENTION MAPPING TO DEVELOP RESOURCES TO PROMOTE SOCIAL ENGAGEMENT WITH HOMEBOUND OLDER ADULTS

**DOI:** 10.1093/geroni/igad104.1395

**Published:** 2023-12-21

**Authors:** Kali Thomas, Kristen Smith, Jennifer Bunker, Kim Gans

**Affiliations:** Johns Hopkins University, Baltimore, Maryland, United States; Brown University, Providence, Rhode Island, United States; Brown University, Providence, Rhode Island, United States; Brown University, Providence, Rhode Island, United States

## Abstract

Homebound older adults are at increased risk of social isolation. Drivers who deliver meals to 800,000+ homebound older adults yearly are uniquely positioned to address social isolation through their conversations and daily interactions. The objective of this study was to develop and evaluate resources to educate meal-delivery drivers about social isolation and promote driver-client social engagement. We used Intervention Mapping (IM), a six-step theoretical- and evidence-based planning approach, to develop this intervention. First, we conducted a needs assessment through two focus groups with drivers and 12 interviews with subject matter experts (Step 1). Our literature review revealed the behavior outcomes, determinants, methods, and strategies to target (Steps 2&3). We piloted the intervention materials with stakeholders, meal-delivery drivers, and subject matter experts (Step 4). Our needs assessment indicated that drivers receive no training or education on social isolation. Drawing on the Social Cognitive Theory and Health Belief Model, we developed a 2.5 minute educational video and website with resources for workers who engage with socially isolated homebound older adults. The video and website included the following change methods and strategies: tailoring, modeling, chunking, cuing, raising, framing, and using imagery to influence behaviors. Feedback from drivers and experts on the resources was positive. The presenter will discuss the results implementing and evaluating the intervention (Steps 5&6) with meal-delivery drivers in Mississippi through an online survey. Our findings indicate the benefits of using an iterative, step-by-step intervention development framework to organize and structure an intervention to address social isolation among homebound older adults.

